# Consanguinity and willingness to perform premarital genetic screening in Sudan

**DOI:** 10.1038/s41431-023-01438-1

**Published:** 2023-08-01

**Authors:** Yasir Ahmed Mohammed Elhadi, Salma S. Alrawa, Esraa S. A. Alfadul, Esra Abdallah Abdalwahed Mahgoub, Austen El-Osta, Safaa Abdalazeem Belal, Don Eliseo Lucero-Prisno, Noha Ahmed El Dabbah, Ashraf Yahia

**Affiliations:** 1 Department of Public Health, Sudanese Medical Research Association, Khartoum, Sudan; 2 Faculty of Medicine, University of Khartoum, Khartoum, Sudan; 3 Department of Community Medicine, Faculty of Medicine, University of Alneelain, Khartoum, Sudan; 4 Self-Care Academic Research Unit (SCARU), School of Public Health, Imperial College London, London, UK; 5 Faculty of Medicine, the National Ribat University, Khartoum, Sudan; 6 Department of Global Health and Development, London School of Hygiene and Tropical Medicine, London, UK; 7 Department of Health Administration and Behavioural Sciences, High Institute of Public Health, Alexandria University, Alexandria, Egypt; 8 Center of Neurodevelopmental Disorders (KIND), Centre for Psychiatry Research, Department of Women’s and Children’s Health, Karolinska Institutet and Stockholm Health Care Services, Region Stockholm, Stockholm, Sweden; 9 Astrid Lindgren Children’s Hospital, Karolinska University Hospital, Solna, Sweden

**Keywords:** Public health, Risk factors

## Abstract

Consanguineous marriage is prevalent in certain world regions due to cultural, economic, and social reasons. However, it can lead to negative consequences including an increased risk of genetic disorders in offspring. Premarital genetic screening (PMGS) is an important tool to identify and manage these risks before marriage. This study aimed to assess the magnitude of consanguineous marriage, knowledge of genetic diseases and PMGS, and attitudes and willingness to perform PMGS in Sudan. A national household survey was conducted using a multistage sampling technique, with a sample size of 2272 participants. Data were collected from December 2022 to March 2023 using an interviewer-administered questionnaire. A significant proportion of respondents (364/850, 42.8%) were married to consanguineal partners, with various types of relatedness. Moreover, 32.1% (242/755) of single respondents were planning to marry a close relative, signifying the likely persistence of consanguineous marriages in Sudan. The level of knowledge regarding genetic diseases and PMGS was relatively low in many states of Sudan, indicating the need for increased awareness interventions. A significant number of participants (85.2%) agreed that premarital screening is effective in reducing genetic diseases, whereas 71.2% supported the introduction of a mandatory PMGS program. Excluding married participants, 82.3% (1265/1537) of respondents were willing to perform PMGS, if implemented. These findings reflect the public positive attitude towards introducing the PMGS program and policies in Sudan and underscore the importance of addressing the knowledge gap of PMGS before such a potential implementation.

## Introduction

Consanguineous marriage is common in certain world regions due to cultural, economic and social reasons [1]. The prevalence of consanguineous marriage varies according to country, region, and population. Nearly one billion individuals globally live in communities that normalize consanguineous marriages, which are most prevalent in the Middle East [1].

Consanguineous marriage can have several negative health consequences, both for the individuals involved and their offspring, particularly when the couple shares a high degree of genetic relatedness [2]. It significantly contributes to women’s adverse reproductive health and fertility behavior, and increases the risk of genetic disorders and the likelihood of inheriting detrimental recessive genetic variants from both parents [3, 4] leading to a higher prevalence of congenital malformations, intellectual disability, and inherited disorders such as thalassemia and sickle cell anemia [4, 5]. Consanguineous marriage can also lead to a higher rate of infant mortality and morbidity [6] as the progeny is more likely to be born with congenital defects and are at a higher risk of developing genetic disorders and chronic diseases later in life [1, 4, 7, 8].

Sudan is located in East Africa and is characterized by ethnically and culturally diverse populations [9]. Hemoglobinopathies, particularly sickle cell disease, are common in Sudan. In one state, the prevalence of sickle cell disease is 2% and the prevalence of sickle cell trait is 25% [10]. Other recessive genetic diseases are also potentially common though their prevalence is unknown due to diagnostic difficulties. Collaborations and the advent of next-generation sequencing enabled the diagnosis of cohorts of Sudanese patients with neurological diseases, e.g., hereditary spastic paraplegia and ataxia [11, 12], leukodystrophy [13], intellectual disabilities [14], and Parkinson’s disease [15], including patients with founder mutations [11, 14, 16]. Also, patients with non-neurological recessive diseases have been reported [17, 18]. Except for isolated reports, the prevalence of consanguinity and/or genetic diseases was not systematically studied in Sudan. One study reported a consanguinity rate of 67% in three towns in western Sudan [10]. Other studies reported a high prevalence of recessive hemoglobinopathies in certain Sudanese populations [19], which could be explained by postulated high consanguinity rates.

Premarital screening (PMS) refers to a set of medical tests that are conducted before marriage to detect and identify genetic, infectious or other medical conditions that could affect the health of the couple or their offspring [20]. The tests in a PMS typically include blood tests for infectious diseases such as HIV and hepatitis B, as well as tests for genetic disorders (PMGS) such as sickle cell anemia, thalassemia and Tay-Sachs disease [21]. The importance of PMS lies in its potential to prevent the transmission of genetic disorders and other health risks from one generation to the next [22]. By identifying and managing these risks before marriage, couples can make informed decisions about their reproductive choices and take steps to minimize the likelihood of passing on genetic disorders to their children [23].

The level of knowledge regarding PMS varies in low and middle income countries (LMICs), particularly in conservative societies where marriage is viewed as a religious or social duty [24]. However, there have been efforts to increase awareness and access to PMS services, with many countries in the Middle East having implemented mandatory PMGS programs for certain conditions such as sickle cell disease and thalassemia [25, 26]. Nonetheless, the utilization of PMS, including PMGS, services can be affected by various factors, including cultural beliefs, attitudes, and socioeconomic factors. In some cultures, marriage is viewed as a private matter that does not involve medical testing [27]. In addition, access to healthcare services and trained healthcare providers may be limited in certain regions or communities, particularly LMICs. Socioeconomic factors such as poverty, lack of education, and gender inequalities can also affect the utilization of PMS services [22]. For example, women may be less likely to seek PMS due to social and economic barriers, including a lack of autonomy and decision-making power within the family, as well as limited financial resources to pay for medical services [28].

PMGS can significantly impact public health by reducing the prevalence of genetic diseases in populations where consanguineous marriage is common [25]. In Sudan, we lack information about the exact prevalence of genetic diseases and consanguinity and the infrastructure of genetic testing. Clinical genetic studies are meager and are mostly collaborative studies between institutions in Sudan and international partners [29, 30]. Genetic testing is not part of the essential health benefits package for primary healthcare in Sudan. It is not available in the public sector, nor the national health insurance, and available only at a few private hospitals and laboratories in the capital city of Sudan. We lack the accurate numbers of those facilities as, apart from a single article on bioinformatics infrastructure [29], no published report explored the infrastructure of genetic testing in Sudan. Also, in our opinion, those knowledge gaps on the prevalence of genetic diseases and consanguinity and the deficient infrastructure of genetic testing are prerequisites that hamper the implementation of PMGS program in Sudan.

This study aimed to assess the knowledge and awareness of community dwelling adults about PMGS, their attitudes and willingness to perform PMGS if implemented in order to help characterize potential barriers and enablers for the development of congruent PMGS program and policies.

## Subjects and methods

This interview-based household survey assessed the magnitude of consanguineous marriage, knowledge of PMGS services, and attitudes toward introducing a mandatory PMGS policy in Sudan. The study was conducted following the Strengthening the Reporting of Observational Studies in Epidemiology (STROBE) statement [31].

### Sampling

The study sample size was calculated using EPI-info-7 software based on an estimated number of Sudanese adults of 19,849,227, 50% expected frequency of consanguineous marriage, alpha error 0.05; design effect equals 2, and an acceptable margin of error of 5%. The sample size was increased by 120% to account for possible non-response and attrition and to account for stratification yielding a minimum required sample size of 1846 participants. The sample was selected using a multistage sampling technique. Sudan has 18 states and 189 localities. Each state was represented by a randomly selected district. The number of study participants was calculated in proportion to the state’s population. In each district, two localities were selected randomly. The selected localities were divided into clusters of 20 households. The number of randomly selected clusters was proportional to the required number to represent each state.

### Interview guide and data collection

An interviewer-administered questionnaire was formulated according to our study objectives and the published literature [27, 32–34]. The questionnaire was divided into four sections, assessing the socio-demographic characteristics, consanguinity, knowledge of genetic diseases and PMGS services, and attitudes and willingness to perform PMGS Consanguineous marriage is defined as a union between two individuals who are related as second cousins or closer [35]. In cases of polygyny, we considered the last partner. The questionnaire was revised for its face validity by members of the Journal Club Group in Sudan, and it was piloted in different states to ensure items’ understandability. The reliability of the subscales used was assessed using the Cronbach Alpha coefficient (0.98). The pilot data was not included in the final analysis. Following comprehensive training, the interviews were conducted by medical students across the Sudanese states, and data was collected using the Kobo-toolbox survey tool, a software developed by Harvard Humanitarian Initiative for data collection in challenging environments [36]. Data were collected from 11 December 2022 to 9 March 2023, with interviews lasting an average of 10 min each.

### Ethical considerations

This study was carried out following the ethical standards outlined in the Helsinki Declaration of 1964 and its subsequent amendments, as well as comparable ethical standards [37]. The study was approved by the University of Gezira, Faculty of Medicine, Health Sector Ethical Review Committee (IRB no: 22–26). The anonymity and confidentiality of the participants were ensured. All participants consented orally to take part in the study. Before data collection, it was made clear that participants could opt out at any time.

### Data analysis procedures

Data from the personal interviews were extracted in an Excel sheet and analysed using the Statistical Package for the Social Sciences software version 26. For the knowledge assessment, the correct answer was given a score of 2, the wrong answer was given a score of 0, and a total knowledge score was calculated for each participant. The score ranged from 0 to 20 points, with higher scores representing higher knowledge. The Kolmogorov–Smirnov test was used to verify the normal distribution of data. The mean score was set as the cut-off point to categorize knowledge data (Score > the mean = good knowledge) and (Score ≤ the mean = poor knowledge). Descriptive statistics were given, and the Independent *T* test and one-way ANOVA test were used to compare categorical demographics and knowledge scores. Spearman’s rank-order correlation was used to assess the correlation between age and knowledge score. The significance was set at the 0.05 level of alpha error.

## Results

### Participant’s characteristics

A total of 2272 participants with a mean age of 29.6 ± 11 years were interviewed. Many of the participants were unemployed (1066/2272, 46.9%), males (1142/2272, 50.3%), and resided in Khartoum state (339/2272, 14.9%). More than half (1422/2272, 62.6%) were single and were educated to the university level (1407/2272, 61.9%). The demographic profile of study participants is shown in Table 1.

**Table 1:** Respondent characteristics (*N* = 2272).

Variables	Categories	*n*	(%)
Gender	Female	1130	(49.7)
	Male	1142	(50.3)
Age (Years)	≤25	1129	(49.7)
	26 to 35	670	(29.5)
	36 to 45	248	(10.9)
	46 to 55	136	(6.0)
	>55	89	(3.9)
State of residency	Khartoum	339	(14.9)
	South Darfur	238	(10.5)
	Al Gezira	226	(9.9)
	West Darfur	156	(6.9)
	North Kordofan	150	(6.6)
	Red sea	127	(5.6)
	Gadarif	122	(5.4)
	Kassala	116	(5.1)
	North Darfur	111	(4.9)
	Central Darfur	109	(4.8)
	White Nile	103	(4.5)
	South Kordofan	96	(4.2)
	Sinnar	86	(3.8)
	River Nile	65	(2.9)
	East Darfur	63	(2.8)
	West Kordofan	62	(2.7)
	Northern	54	(2.4)
	Blue Nile	49	(2.2)
Education	Illiterate	75	(3.3)
	Primary school	171	(7.5)
	Secondary school	379	(16.7)
	University	1407	(61.9)
	Informal education	47	(2.1)
	Postgraduate degree	193	(8.5)
Employment	Free worker	612	(26.9)
	Governmental employee	405	(17.9)
	Employee in the private sector	189	(8.3)
	Unemployed	1066	(46.9)
Marital status	Single	1422	(62.6)
	Married	735	(32.4)
	Widowed	46	(2.0)
	Divorced	69	(3.0)

### Prevalence of consanguineous marriage in Sudan

**Fig. 1:**
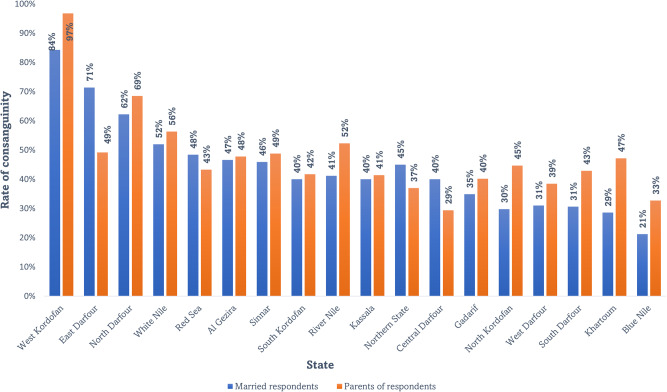
Rate of consanguine marriage in Sudanese states, 2022. The rates of consanguine marriage among parents of respondents and married participants were high in the western regions of the country with the highest magnitudes reported in West Kordofan, East Darfur, and North Darfur states.

Excluding single respondents, participants were asked about the extent that they are related to their partner, if at all. A significant proportion of respondents (364/850, 42.8%) were married to a relative, among whom 44.5% (162/364) were related paternally, 28.6% (104/364) maternally, whereas 26.9% (98/364) were related from both parents’ sides. Nearly half (1058/2272, 46.6%) of respondents were born to consanguineous parents. Half of the single respondents (755/1537, 49.1%) were planning to marry; among those, 32.1% (242/755) were considering a consanguineal partner (Table 2). The rates of consanguine marriage among parents of respondents and marriage participants were high in the western regions of the country with the highest magnitudes reported in West Kordofan, East Darfur, and North Darfur states (Fig. 1).

**Table 2:** Consanguinity among participants’ parents, married respondents, and prospective couples.

X	Variables	Categories	*n*	(%)
Married	Partner Consanguinity	Relative*	364	(42.8)
		Not relative, but same tribe	161	(18.9)
		Not relative, not similar tribe	325	(38.3)
		Total	850	(100.0)
	Type of relatedness	From father side	162	(44.5)
		From both father and mother sides	98	(26.9)
		From mother side	104	(28.6)
		Total	364	(100.0)
Parents	Parents’ consanguinity	Relative*	1058	(46.6)
		Not relative, but same tribe	518	(22.8)
		Not relative, not similar tribe	696	(30.6)
		Total	2272	(100.0)
	Type of relatedness	From father side	423	(40.0)
		From both father and mother sides	386	(36.5)
		From mother side	249	(23.5)
		Total	1058	(100.0)
Prospective couples	Planning to marry	No	782	(50.9)
		Yes*	755	(49.1)
		Total	1537	(100.0)
	Potential partner’s relatedness	Relative	242	(32.1)
		Not relative, but same tribe	115	(15.2)
		Not relative, not similar tribe	398	(52.7)

*X* Population.

*Further assessment of type of relatedness provided below.

### Knowledge, attitudes and willingness to perform premarital genetic screening

The mean knowledge score among participants was 8.95 ( ± 3.89 S.D), and the maximum score achieved was 20. More than half of the participants 53.6% (1218/2272) had good knowledge about genetic disorders and PMGS while many of the participants (1054/2272, 46.4%,) showed poor levels of knowledge (Table 3).

**Table 3:** Factors associated with knowledge of genetic diseases and PMGS in Sudan.

Variables			%(*n*)
The total score of participants’ knowledge		Good knowledge	53.6 (1218)
		Poor knowledge	46.4 (1054)
		Total knowledge score (Mean)	*p*
Gender	Female	9.0	0.685
	Male	8.9	
Education	Illiterate	5.1	<0.001
	Primary school	5.8	
	Secondary school	8.6	
	University	9.6	
	Informal education	5.2	
	Postgraduate degree	10.2	
Employment	Free worker	8.2	<0.001
	Not working	9.2	
	Governmental employee	9.1	
	Employee in the private sector	10.0	
Marital status	Single	9.5	<0.001
	Widowed	6.0	
	Married	8.2	
	Divorced	8.0	
Willingness to perform PMGS	Yes	9.7	<0.001
	No	8.0	
	Not applicable	6.0	

**Fig. 2:**
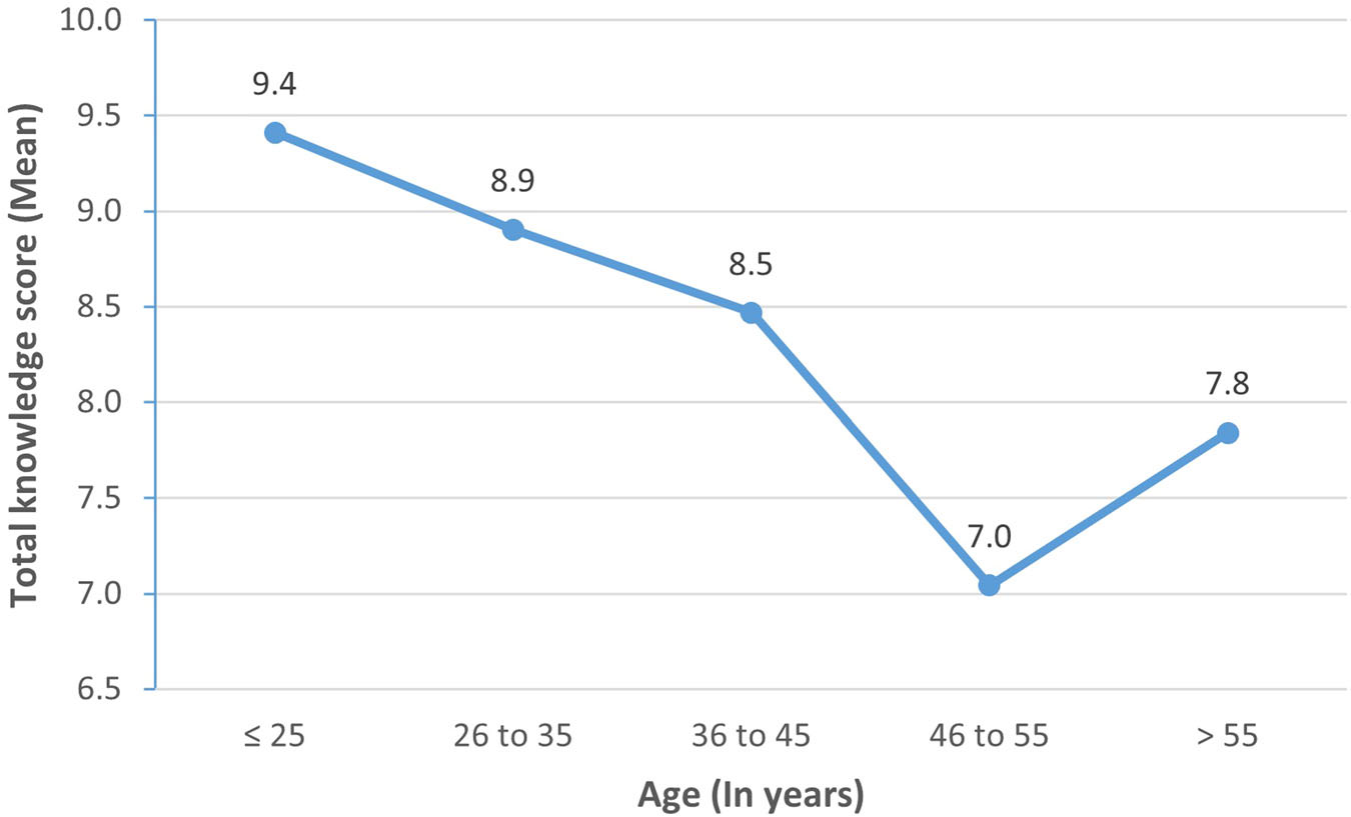
Participants’ total knowledge scores (mean) by age groups (*N* = 2272). Spearman’s rank-order correlation revealed a negligible, negative correlation between participants’ age and total knowledge score (*r*_*s*_ = −0.130, *p* = 0.001).

Spearman’s rank-order correlation revealed a negligible, negative correlation between age and total knowledge score (*r*_*s*_ = −0.130, *p* = 0.001). The highest mean knowledge score was among participants aged ≤25 years and the lowest was reported among those aged 46 to 55 years (Fig. 2).

**Fig. 3:**
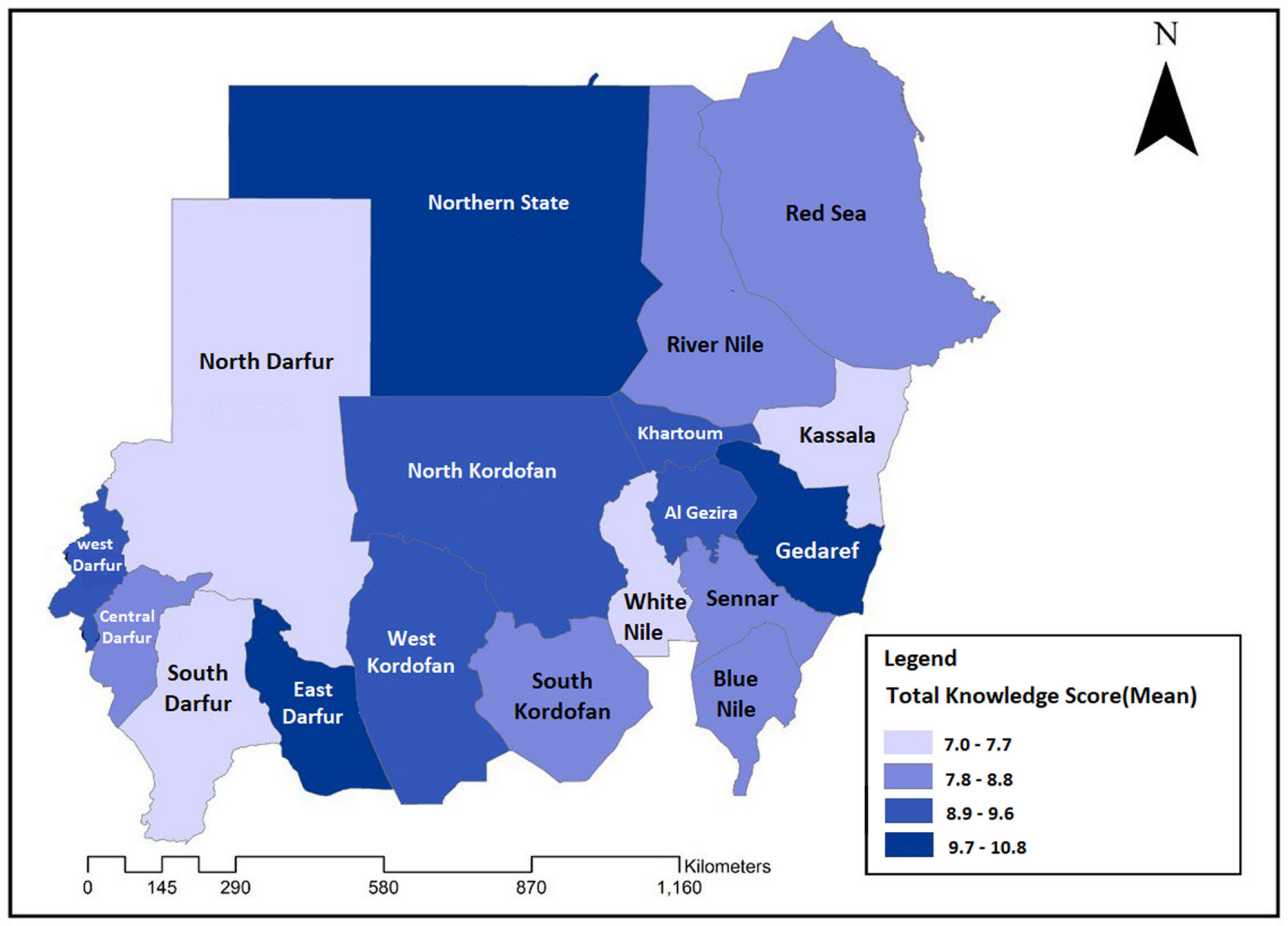
Sudan map showing participants’ total knowledge scores (mean) by states (*N* = 2272). A statistically significant difference in the knowledge score was observed between participants from different states (*p* < 0.001).

There was no significant difference in the total knowledge score between males and females (*p* = 0.685). However, a statistically significant difference in the knowledge score was observed between participants from different states, educational levels, employment statuses, and marital statuses (*p* < 0.001). Candidates with a postgraduate degree, employees in the private sector, single participants, and those willing to perform PMS if available, had higher knowledge scores than their counterparts (Table 3). Moreover, participants from the Northern state, East Darfur, Gadarif, and Khartoum states had the highest mean total knowledge scores, whereas participants from Kassala, White Nile and the North Darfur states demonstrated the lowest knowledge scores (Fig. 3). However, there was no significant correlation between the knowledge score and the rate of consanguinity at the state level.

Most participants (1935/2272, 85.2%) agreed that PMS would likely be effective in reducing genetic diseases, and 71.2% (1617/2272) agreed that premarital testing should be mandatory for all Sudanese population. Excluding married participants, most respondents (1265/1537, 82.3%) were willing to perform PMS for genetic disorders. In cases of a positive PMGS result, 35.9% (552/1537) thought their decision will depend on the nature and severity of the disease, and 27% (415/1537) indicated that they will marry another partner (Table 4).

**Table 4:** Participants’ attitude and willingness to perform premarital genetic screening.

Variables		Strongly Agree % (*n*)	Agee % (*n*)	Neutral % (*n*)	Disagree % (*n*)	Strongly disagree % (*n*)
Premarital screening is effective in reducing genetic diseases		40.0(908)	45.2(1027)	7.6(172)	6.3(142)	1.0(23)
Premarital screening should be mandatory for all Sudanese population		30.8(700)	40.4(917)	11.8(269)	14.6(331)	2.4(55)
Premarital screening should be mandatory for specific groups in Sudan		8.2(186)	19.1(434)	13.9(315)	43.4(986)	15.4(351)
Variables					*n*	(%)
Willingness to perform PMGS	Yes				1265	(82.3)
	No				209	(13.6)
	Not applicable				63	(4.1)
	Total				1537	(100.0)
Action in case of a positive screening results	I will marry my partner regardless of consequences				404	(26.3)
	Not getting married at all				166	(10.8)
	According to the probability of disease				552	(35.9)
	I will not marry this partner, but I can marry someone with compatible results				415	(27.0)
	Total				1537	(100)

## Discussion

This study provides valuable insights into consanguineous marriage and the public preparedness for PMGS practices in Sudan and highlights the need for a comprehensive public health strategy to address genetic disorders and improve reproductive health outcomes. To our knowledge, this is the first nationwide study to explore the magnitude of consanguineous marriage, knowledge of genetic diseases and PMGS, and to explore the attitudes and willingness of community dwelling adults to participate in PMGS programs in Sudan.

Our findings revealed a high prevalence of consanguineous marriage among married participants (364/850, 42.8%), parents of respondents (1058/2272, 46.6%), and prospective couples (242/755). As is the case with other Middle Eastern countries, consanguineous marriage is a deeply ingrained cultural practice in Sudan. One reason for this high prevalence is related to the cultural value placed on family and maintaining familial ties, since “*marrying within the family*” is seen as a way to strengthen family ties and maintain family honor [38, 39]. Another reason stems from the belief that marrying within the family will ensure the compatibility of the couple and that the union would lead to a stable and long-lasting marriage [8]. Poverty, lack of education and ethnicity can also limit an individual’s options for finding a suitable marriage partner, and this could lead to consanguineous marriages. Although the burden of genetic diseases in Sudan has not been formally reported, the remarkably high consanguinity rates recorded in our study coupled with the increasing number of isolated reports of recessive genetic disorders [11, 18, 19] highlight a pressing need to raise awareness about, and assess the prevalence of genetic diseases in Sudan so that they may be addressed.

A significant proportion of participants in the current study demonstrated poor levels of knowledge regarding genetic diseases and PMGS services, highlighting the need for educational interventions. This lack of knowledge about genetic disorders and their link to consanguineous marriage is striking but unsurprising because PMS is not currently prioritized as a public health intervention in Sudan. Establishing a national PMGS service would require significant investment and infrastructure including services, policies and frameworks to drive implementation. In many parts of Sudan, health education programs and campaigns are often limited or absent, and people may not have access to reliable sources of information. Hence, many people are not aware of the importance and benefits of PMGS. More importantly, people with genetic or infectious diseases may face stigma and discrimination in some societies [40], discouraging them from seeking PMS or disclosing their conditions to potential partners. Poor knowledge of PMGS is not peculiar to Sudan as studies in culturally-related populations have shown similar patterns [41, 42]. Addressing these challenges requires a multi-faceted approach, including health education and awareness campaigns, health systems strengthening, access to services, promoting cultural sensitivity and inclusivity, and the development and implementation of supportive policies and programs. It will also be crucial to assess the burden of different genetic diseases in Sudan to inform the development of a congruent PMGS program that prioritizes the most common genetic diseases in each region.

That the knowledge score was significantly associated with the state of residency, age, educational level, employment status, and marital status of participants could be explained by differences in access to knowledge platforms among these entities. This finding is consistent with other studies which showed that knowledge of PMS was associated with respondents’ age, level of education and occupation [43, 44]. These factors should be considered when developing target-oriented educational interventions to increase awareness of the benefits and importance of PMS.

There was a positive attitude towards the effectiveness of PMS in reducing genetic diseases, with the majority of respondents agreeing that PMGS should be mandated in Sudan, and that they would be happy to partake in genetic screening. Indeed, Mandating PMS could help promote healthy families and reduce the burden of genetic disorders and infectious diseases [45, 46]. However, implementing such a policy could raise concerns about personal privacy, autonomy and discrimination [47, 48]. It is also important to consider the potential consequences of positive screening results, including stigmatization and the impact on the couple’s decision to marry. In the current study, a quarter of participants were willing to marry their consanguineous partners regardless of the consequences. While some studies showed that PMS is effective in reducing at-risk marriages [21], more recent studies suggest that PMS and genetic counseling programs were largely unsuccessful in discouraging at-risk marriages [25, 49]. Whilst the development and large-scale implementation of a national PMS policy is desirable, it should be based on a comprehensive assessment that takes into account various factors including benefits and risks, as well as costs, cultural, ethical and legal factors [50]. It is also necessary to involve relevant stakeholders, including healthcare professionals, policymakers, and community leaders in the decision-making process to ensure that the policy is sensitive to local contexts and reflects the values and needs of the population.

### Limitations

This study encountered several limitations. Adding to the inherent limitations of the cross-sectional research design, we did not include many socio-economic variables to avoid having a lengthy interview. Most respondents were educated at a university level. This introduces a risk of selection bias by potentially overlooking valuable insights from individuals with other educational backgrounds. Also, the number of married respondents was relatively low, and the sample was slightly biased toward single participants. Accordingly, the rates of consanguinity may have been underestimated among the respondents. However, the overall consanguinity rates among the married respondents (364/850, 42.8%) is comparable to the parents’ group (1058/2272, 46.6%). These limitations may limit the generalizability of the results to all Sudanese population. Furthermore, the findings of the current study are prone to social desirability bias having relied on a self-reporting assessment.

## Conclusion

The remarkably high prevalence of consanguineous marriages in Sudan highlights the need for a comprehensive public health strategy to address genetic disorders and improve reproductive health outcomes. Addressing the knowledge gap and promoting PMS services in Sudan is crucial and calls for the development of the implementation of a public health strategy to raise awareness about the risks associated with consanguineous marriage and the benefits of PMGS. Efforts should be made to provide comprehensive genetic counseling services, improve access to healthcare facilities and upskill the healthcare workforce so it is better able to deliver the service alongside coherent public health messaging concerning consanguineous marriage. Community-based interventions, including educational and awareness campaigns, should be implemented to challenge cultural norms and beliefs that contribute to the adverse outcomes of consanguineous marriage.

## Supplementary information

STROBE Statement

## Data Availability

All data supporting the findings of the current study are available from the corresponding author upon reasonable request.
